# The Prevalence of Hepatic Steatosis in Patients With Normal Coronary Computed Tomography Angiography: Is It Time for a Paradigm Shift?

**DOI:** 10.7759/cureus.102737

**Published:** 2026-01-31

**Authors:** Marc T Zughaib, Chad Nicholson, Vincent Lipari, Hardik Patel, Marcel E Zughaib

**Affiliations:** 1 Cardiology, Ascension Providence Hospital - Michigan State University College of Human Medicine (MSUCHM), Southfield, USA; 2 Cardiology, Henry Ford Providence Hospital, Southfield, USA; 3 Cardiology, Hartford Hospital, Hartford, USA; 4 Cardiology, Kaiser Permanente Fresno Medical Center, Fresno, USA

**Keywords:** calcification, cardiac imaging, cardiology, coronary cta, hepatic steatosis

## Abstract

The role of coronary computed tomography angiography (CCTA) in diagnostic cardiology is steadily increasing. Normal CCTAs successfully reclassify patients to a low-to-intermediate risk category with marked reduction in cardiovascular events. However, hepatic steatosis has been associated with adverse cardiovascular events and can be routinely evaluated on non-contrast CCTAs. The prevalence of hepatic steatosis in the setting of normal CCTAs is not known and may be of clinical significance.

We conducted a retrospective, single-center analysis to evaluate for the presence and incidence of hepatic steatosis in the setting of normal CCTAs. Non-contrast images were used to identify/measure the presence or absence of hepatic steatosis. The presence of hepatic steatosis was defined based on attenuation measurements in Hounsfield units (HU), by comparing the liver and spleen.

Of 297 patients screened, a cohort of 99 patients were identified with a coronary calcium score of zero. Ten patients (10%) were found to have non-calcified plaque on CCTA and were excluded from the study population. Of the remaining 89 patients, 13 patients (14%) were found to have evidence of hepatic steatosis.

Patients with normal CCTAs are often reassured, supported by data predicting an improved prognosis with low rates of cardiovascular events. We propose that evaluating and identifying patients with underlying hepatic steatosis, a known associated marker of atherosclerosis, even in patients with "normal" CCTAs is important, as this correlates with higher cardiovascular risk.

## Introduction

Coronary artery calcium (CAC) scoring detects subclinical atherosclerosis, which independently and strongly predicts future coronary heart disease (CHD). When used in low-to-intermediate-risk patients, the coronary calcium score is a strong predictor of incident CHD and provides predictive information beyond that provided by standard risk factors in four major racial and ethnic groups in the United States [1]. Low CAC scores are associated with a low risk, whereas high CAC scores are associated with a worse event-free survival.

Coronary computed tomography angiography (CCTA) adds further diagnostic value to the CAC score by providing an accurate, non-invasive anatomic evaluation of the coronary anatomy. Newer scanners with improved imaging protocols have improved spatial and temporal resolution with an estimated 78% reduction in radiation exposure over the past 10 years [2]. Given the high diagnostic accuracy with sensitivity of 98%-99% and low radiation dose [3], CCTAs are being suggested as a possible first-line diagnostic test in the evaluation of coronary artery disease.

Normal CCTAs, defined by normal coronary anatomy with no coronary calcification and no evidence of positive or negative vascular remodeling, predict an excellent prognosis. In a recent study, cumulative event-free survival was 100% with a mean follow-up of 52 months for hard and all events in patients with normal coronary arteries on CCTA [4]. However, there may be a subset of patients who are at higher risk for CHD, despite having normal coronary CCTAs.

Non-alcoholic fatty liver disease (NAFLD), the most common chronic liver disease in the United States, extends across a spectrum starting from hepatic steatosis, progressing to nonalcoholic steatohepatitis (NASH), and culminating in cirrhosis. In the United States, studies report a prevalence of NAFLD of 10%-46%, with most biopsy-based studies reporting a prevalence of NASH of 3%-5% [5]. NAFLD shares many similar risk factors with coronary artery disease (CAD), including central obesity, type 2 diabetes mellitus, dyslipidemia, and metabolic syndrome; and, to no surprise, a strong established correlation exists between NAFLD and CAD. A systematic review and meta-analysis of 34 studies showed that NAFLD was associated with an increased risk for incident cardiovascular disease (CVD), with a hazard ratio (HR) of 1.37, and specifically an increased risk for CAD (HR, 2.31) and hypertension (1.16) when compared with patients without NAFLD [6]. A large prospective cohort study by Wong et al. revealed a higher prevalence of coronary artery stenosis (84.6% versus 64.1%; P < 0.001) and need for percutaneous coronary intervention (68.3% versus 43.4%; P < 0.001) in patients with NAFLD versus without NAFLD [7]. A meta-analysis of 16 cohort studies with a median seven-year follow-up revealed that patients with NAFLD were 64 times more likely than patients without NAFLD to have fatal or nonfatal cardiovascular events, such as myocardial infarction, stroke, angina, or coronary revascularization [8]. In addition, cardiovascular disease has been established as the most common cause of mortality in patients with NAFLD [9].

Although not routinely performed, the presence or absence of hepatic steatosis can easily be estimated and calculated from non-contrast CT images and may provide useful supplemental information to further risk-stratify patients undergoing CCTAs. Hepatic steatosis, despite normal CCTAs, may procure a higher independent cardiovascular risk. Our study aimed to evaluate the rate of occurrence of NAFLD - as defined by the presence of hepatic steatosis on non-contrast CT images - to possibly identify a subgroup of patients who may be at higher cardiovascular risk despite normal CCTAs.

## Materials and methods

**Figure 1:**
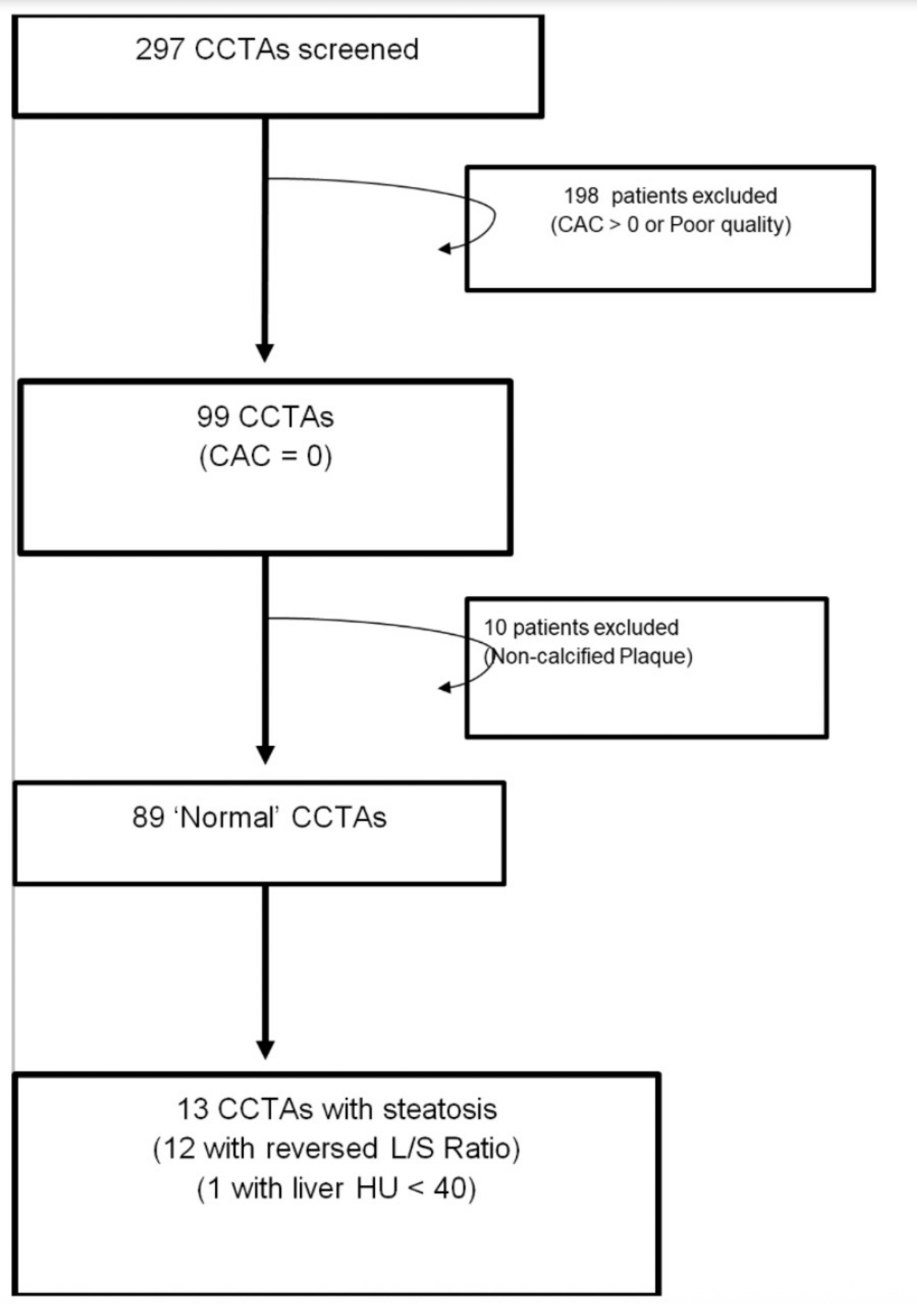
Flowchart illustrating patient selection CCTA: Coronary computed tomography angiography; CAC: Coronary artery calcium; L/S: Liver/spleen.

This study is a retrospective, single-center analysis aimed to evaluate the presence of hepatic steatosis in the setting of normal CCTAs. The study protocol was approved by the local Institutional Review Board (IRB approval ID: 1541180-1). We reviewed CCTAs performed at Ascension Providence Hospital, Southfield, from January 1 to October 31, 2019, using the GE Revolution CT scanner with 160 cm whole organ coverage, 0.23 mm resolution, and 437 mm/s table speed. CCTAs were performed/acquired using the standard protocol with adequate heart rate control (target < 65 beats per minute), sublingual nitroglycerin, and prospective gating. Image quality was determined by the reader and documented in the report. Poor-quality studies were excluded from the study cohort. Subsequently, a cohort of patients with zero coronary calcium and no significant non-calcified CAD (i.e., essentially “normal” coronary computed tomography (CCT)) was identified (Figure 1).

The non-contrast images used to acquire the calcium score were used to identify the presence or absence of hepatic steatosis based on a well-documented and validated assessment of hepatic steatosis on non-contrast CT. Specifically, the attenuation of the liver measured in Hounsfield units (HU) is normally higher than that of the spleen (L/S > 1). If the ratio is reversed (L/S < 1), this is highly suggestive of fatty liver [10,11]. Also, if the liver HU attenuation is <40, the liver fat content is probably >30% [12].

**Figure 2:**
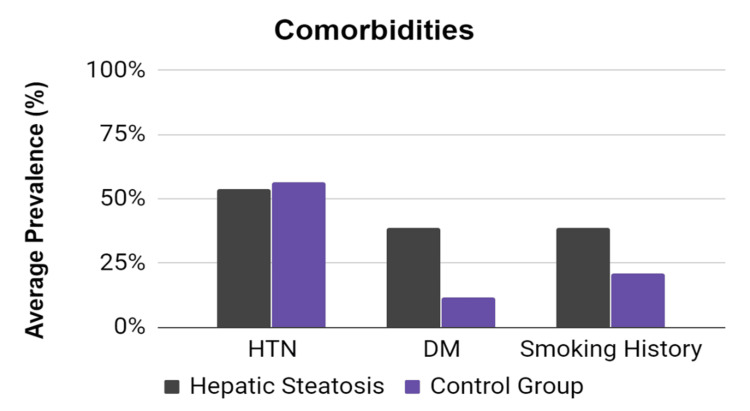
Prevalence of comorbidities in patients with and without hepatic steatosis DM: Diabetes mellitus; HTN: Hypertension.

The protocol proposed by the MESA (multi-ethnic study of atherosclerosis) study was used to determine the presence of fatty liver from CT CAC scoring acquisitions. Using a region of interest (ROI) > 100 mm^2^ area, three regions of interest were measured: two in the right lobe of the liver (one anterior to the other) and one ROI in the left lobe of the liver. At least two regions of interest were measured in the spleen (Figure 2). Caution was exercised to exclude non-uniform areas, including those of blood vessels. Calculation of the L/S HU ratio was obtained by taking the mean of the liver ROIs and then dividing by the mean of the spleen ROIs. Data were recorded and organized on an encrypted Excel data sheet with the calculation of simple statistical variables. Measurements and calculations were performed by a single reader to ensure consistency throughout the analysis.

Study population

The studied population includes patients of both sexes who have had a CCTA ordered and performed for various, appropriate diagnostic indications. Inclusion criteria were an age of 18-85 years, absence of coronary calcium, and CCTA reported as normal with no calcified or non-calcified plaque (i.e., essential "normal" studies). Key exclusion criteria were a calcium score greater than zero or a calcium score of zero with the presence of non-calcified plaque on CCTA consistent with a diagnosis of coronary artery disease. Poor-quality studies and studies performed for congenital or structural heart diseases were also excluded.

Data analysis

Descriptive statistics such as means, standard deviation (SD), frequencies, and percentages were generated. An independent t-test analysis was conducted to compare the hepatic steatosis group with a control group based on continuous variables. Chi-square test was performed on categorical variables. SPSS version 23 (IBM SPSS Statistics for Windows; IBM Corp., Armonk, NY) was used to analyze the data. Statistical significance was set at p < 0.05.

## Results

Of 297 patients screened, a cohort of 99 patients was identified with a coronary calcium score of zero. In this cohort, 10 patients were found to have non-calcified plaque on CCTA, as evidenced by positive or negative vascular remodeling, consistent with a diagnosis of CAD, and were excluded from the study population. The remaining 89 patients included 31% men (mean (± SD) age, 54 ± 13). Of those, 13 patients (14%) were found to have evidence of hepatic steatosis (46% men; mean, 56 ± 10).

**Figure 3:**
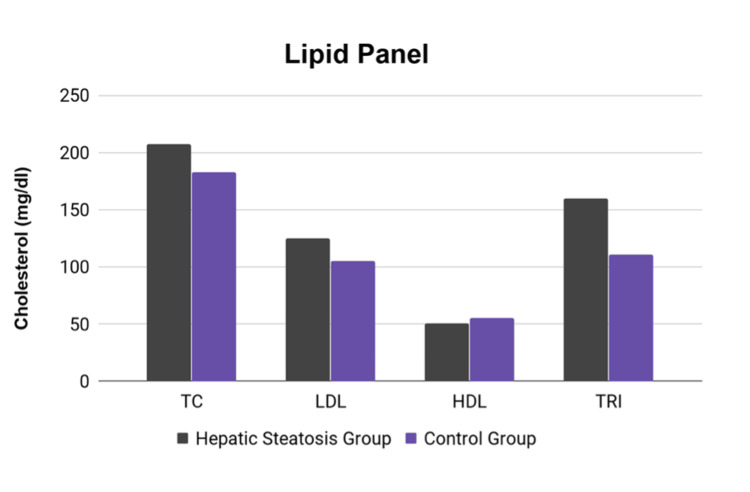
Comparison of lipid panel parameters between the hepatic steatosis and control groups TC: Total cholesterol; LDL: Low-density lipoprotein; HDL: High-density lipoprotein; TRI: Triglycerides.

Baseline characteristics, including age, BMI, and sex, were fairly well-balanced between the groups (Table 1). However, patients with hepatic steatosis on average had a higher incidence of diabetes, a higher rate of tobacco use (Figure 2), and elevated triglycerides (Figure 3). Total cholesterol and LDL cholesterol were higher in patients with hepatic steatosis, with a p-value approaching (but not reaching) statistical significance.

**Table 1:** Baseline characteristics LDL: Low-density lipoprotein; HDL: High-density lipoprotein.

Variables	Hepatic Steatosis (n = 13)	Control (n = 76)	P-value
Age (mean ± SD)	56 ± 11	54 ± 13	
Male (%)	6 (46%)	22 (30%)	0.25
Body-mass index (mean ± SD)	32.1 ± 6	31.3 ± 7	0.69
Diabetes mellitus (%)	5 (38%)	8 (12%)	0.01
Hypertension (%)	7 (54%)	39 (57%)	0.83
Smoking (%)	5 (38%)	14 (21%)	0.21
Total cholesterol (mean ± SD)	207 ± 32	182 ± 35	0.08
LDL (mean ± SD)	124 ± 27	105 ± 27	0.08
HDL (mean ± SD)	52 ± 9	55 ± 14	0.39
Triglycerides (mean ± SD)	160 ± 82	111 ± 57	0.047

**Figure 4:**
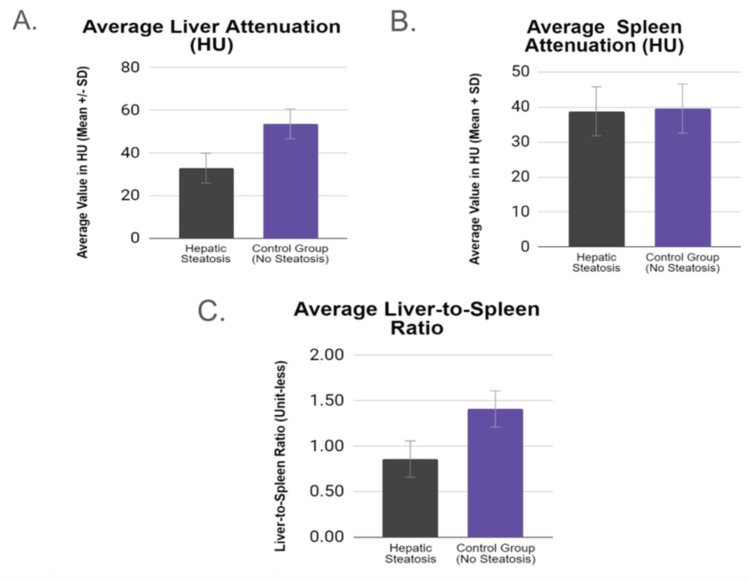
(A) Average liver attenuation, (B) average spleen attenuation, and (C) average liver-to-spleen ratio in patients with hepatic steatosis compared with the control group

The average liver attenuation measured in HU was significantly higher in the group with steatosis (p < 0.005). The liver-to-spleen ratio was significantly lower in the group with hepatic steatosis (p < 0.005). This was expected considering the diagnostic criteria for defining hepatic steatosis that rely predominantly on low liver attenuation and reversal of the liver-to-spleen ratio (Figure 4).

## Discussion

Hepatic steatosis is an early phenomenon on a larger spectrum that extends from fatty liver to steatohepatitis to cirrhosis. A strong correlation exists between non-alcoholic hepatic steatosis and cardiovascular disease, but steatosis can be affected by multiple other variables, including alcohol consumption, hypothyroidism, starvation, hepatotoxic medications, viral hepatitis (B or C), and HIV. This study was performed in a retrospective manner using a computerized patient database. Per our chart review, no patients had any history of hepatitis B, hepatitis C, or other known liver disease processes. The majority of patients with hepatic steatosis denied alcohol use. Three reported social alcohol use, and one reportedly had excessive alcohol use. In addition, social/recreational use of alcohol in the days/weeks leading up to the CT scan could not be assessed accurately.

Non-alcoholic fatty liver disease shares many similar risk factors with CAD, including central obesity, type 2 diabetes mellitus, dyslipidemia, and metabolic syndrome, with a strong established correlation between NAFLD and CAD. In recent years, CCTAs have gained popularity and are increasingly becoming the preferred first-line non-invasive diagnostic test to evaluate for CAD. A normal CCTA with no calcified plaque and no evidence of positive or negative vascular remodeling predicts a very favorable five-year prognosis with 100% cumulative event-free survival at 52 months follow-up ± 22 months for hard events [4]. Our current study showed there may be a significant subgroup of patients who, despite having normal CCTAs, may be at higher future cardiovascular risk due to the presence of an additional cardiovascular risk factor such as hepatic steatosis.

In a recent substudy of the SCOT-HEART trial, NAFLD was independently associated with the presence of CAD and elevated BMI, and patients with potential NAFLD were more likely to have non-obstructive CAD (45% vs 33%, p = 0.003) but not obstructive CAD (29% vs. 24%, p = 0.165) [13]. In addition, several prior studies have expanded on the correlation between NAFLD and dyslipidemia, showing hepatic steatosis correlated with higher serum triglycerides, lower HDL cholesterol [14], and higher LDL cholesterol [15,16]. Our current study reproduces similar results and re-establishes the well-validated relationship between NAFLD, BMI, and dyslipidemia. By only including patients with normal CCTAs, this study elaborates on the previously established relationships and identifies a significant subgroup of patients who have hepatic steatosis despite normal CCTAs, who may be at heightened future risk for the development of CAD.

Evaluation for the presence of hepatic steatosis can be performed quickly and without difficulty from non-contrast images, which are routinely collected and included in the image datasets of CCTAs performed at most institutions. Identifying and reporting this additional cardiovascular risk factor in low-intermediate risk patients who have otherwise normal CCTAs adds useful, additional information that may ultimately influence management options and help optimize the cardiovascular risk profile of these patients long before the onset of clinical cardiovascular disease.

In this study, hepatic steatosis was found in 14% of patients with normal CCTAs. There has been a steady increase and continued projected growth for the use of coronary CTs in the field of cardiology due to an abundance of data illustrating its heightened efficiency [17], cost-effective nature [18], high negative predictive value, and sensitivity [19] for detecting CAD.

Conventionally, following normal coronary CTs, patients are re-classified as low risk and often reassured of an optimal cardiovascular prognosis. Based on our findings, we predict there exists a significant subgroup (~10%-15%) of patients with "normal" coronary CTs that may be falsely reassured. A "negative" CCTA may not be so negative in the presence of hepatic steatosis and traditional cardiovascular risk factors like diabetes, smoking, and hyperlipidemia.

The majority of the patients included in our study were females, comprising approximately 70% of the studied cohort, with an average age of 54 years. This observation may illustrate the increasing use of non-invasive CCTA in the low-to-intermediaterisk, younger female population presenting with atypical CAD symptoms. The preferential use of the CCTA in this population is supported by data from the CRESCENT trial, which illustrated that CCTA is more efficient compared to functional testing in women than in men in terms of time to reach the final diagnosis and avoidance of further unnecessary downstream testing [20]. A predominantly female cohort may bias our results toward the female sex, but it clearly illustrates the added value of identifying the presence of hepatic steatosis in this population. Future studies are needed to see if our findings can be extrapolated equally to both sexes.

Our study is limited by being a single-center retrospective study. The finding may be more beneficial as hypothesis-generating rather than causal. A future, larger study that follows these patients over time to monitor for hard cardiovascular endpoints may help further clarify the importance of diagnosing hepatic steatosis in this subgroup/population.

## Conclusions

Patients with normal CCTAs are often reassured, supported by data predicting improved prognosis with low rates of cardiovascular events. However, we propose that evaluating and identifying underlying hepatic steatosis - a known associated marker of atherosclerosis - even in patients with "normal" CCTAs, may identify a subgroup of patients at a higher cardiovascular risk.
